# Did Kant suffer from misophonia?

**DOI:** 10.3389/fpsyg.2024.1242516

**Published:** 2024-02-14

**Authors:** Arnaud Norena

**Affiliations:** Centre de recherche en Psychologie et Neuroscience, UMR7077, Aix-Marseille Université, CNRS, Marseille, France

**Keywords:** conditioning, anorexia, asceticism, cognitive sciences, social psychology, phenomenology

## Abstract

Misophonia is a disorder of decreased tolerance to specific sounds, often produced by humans but not always, which can trigger intense emotional reactions (anger, disgust etc.). This relatively prevalent disorder can cause a reduction in the quality of life. The causes of misophonia are still unclear. In this article, we develop a hypothesis suggesting that misophonia can be caused by a failure in the organization of the perceived world. The perceived world is the result of both the structure of human thought and the many conditioning factors that punctuate human life, particularly social conditioning. It is made up of abstract symbols that map the world and help humans to orient himself in a potentially dangerous environment. In this context, the role of social rules acquired throughout life is considerable. Table manners, for example, are a set of deeply regulated and controlled behaviors (it’s considered impolite to eat with the mouth open and to make noise while eating), which contribute to shape the way the perceived world is organized. So it’s not surprising to find sounds from the mouth (chewing etc.) among the most common misophonic sound triggers. Politeness can be seen as an act of obedience to moral rules or courtesy, which is a prerequisite for peaceful social relations. Beyond this example, we also argue that any sound can become a misophonic trigger as long as it is not integrated into the perceived ordered and harmonious world, because it is considered an “anomaly,” i.e., a disorder, an immorality or a vulgarity.

## Introduction

### Definition

Misophonia can be defined as a disorder as its associated sound-triggered reactions impose substantial impairment in functioning and/or psychological distress. In brief, misophonia is a disorder of decreased tolerance to specific sounds (Swedo et al., 2022). Misophonia is however not recognized as a disorder in any medical or clinical classification system (e.g., ICD, DSM). These sounds are generally produced by Humans and are often associated with the acts of eating, breathing, coughing, expectorating and/or blowing one’s nose but not always: animal vocalizations (barking) or the sound of objects (fridge noise) can also be misophonic triggers.

The term misophonia (*miso*: hatred, *phonia*: sound) was coined by Jastreboff in the early 2000s (Jastreboff and Jastreboff, 2001; Jastreboff and Jastreboff, 2002; Jastreboff and Jastreboff, 2023). Misophonia is sometimes classified as a sub-category of hyperacousis (Tyler et al., 2014). But while hyperacusis may be defined as a disorder of tolerance of sounds, we feel that the nature of these two disorders is not the same. Hyperacusis may perhaps be defined as a disorder of tolerance to a specific sensorial attribute of a sound, that is the loudness (subjective intensity): the sound is disturbing because its loudness is abnormally and uncomfortably high (Enzler et al., 2021a). In contrast, misophonia is not directly linked to the loudness. As we shall see later in more details, we hypothesize that misophonia is related less to the acoustic properties of the stimulus than to the sound being caused by the behavior of the person responsible, that is to say an inappropriate behavior.

### Reactions, impact, and quality of life

Misophonia can be associated with strong and uncontrollable negative emotions, such as anger or disgust, which may also be accompanied by extreme physical reactions of the ‘fight or flight’ type (shouting, threats, flight, etc.). The misophonic sounds trigger a reaction of the autonomic nervous system, attested by an increase in the cutaneous conductance (Edelstein et al., 2013; Kumar et al., 2017) and an increase in the heart rate (Kumar et al., 2017; Schröder et al., 2019) (but see Siepsiak et al., 2022). A misophonic sound that is very slight or barely audible above the background noise is enough to trigger physical and emotional reactions. As soon as the misophonic sound is perceived, the subjects become hyper-vigilant to the acoustic environment and it is impossible for them to ignore the issue (Edelstein et al., 2013). The misophonic sound can be felt as an aggression and it is for this reason that it mobilizes the organism’s alarm and defence system. Subjects thus lose control of their cognitive resources; they can do nothing but be hypervigilant for the misophonic sounds. Misophonic disorder is comparable to a situation of stress which it is difficult to escape from because the sound cannot be avoided. The auditory system is indeed a system of vigilance (it is the organism’s “radar system”) that catches the attention when a significant sound event occurs. Some subjects, then, has no choice but aggression (i.e., anger, verbal critics or complaints) to stop the person responsible for the behavior, or flight (escape).

Patients are disturbed or destabilized by this loss of control they experience. The experience is so powerful and negative that patients end up avoiding environments (restaurants, cafeterias, cafés, etc.) which might expose them to this disagreeable situation. Misophonic subjects are thus deprived of social events which they might have wished to take part in (dinners with the family, having a drink with friends, etc.). The reactions of aggression toward people who are close and loved ones contributes to the impact of misophonia inasmuch as the situation arouses feelings of sadness and guilt. The loss of control felt by misophonic subjects is also the source of a feeling of powerlessness.

### Assessment and prevalence

Several questionnaires have been developed over the past few years to study and assess misophonia (Schröder et al., 2013; Wu et al., 2014; Siepsiak et al., 2020a; Rosenthal et al., 2021; Vitoratou et al., 2021). Our team has recently developed a psychophysical approach for the diagnosis of misophonia. The test consists for the subjects of rating the sounds on a pleasant to unpleasant visual analog scale (Enzler et al., 2021b). This study shows that misophonic subjects had a normal degree of sensitivity to non-misophonic sounds. This result corroborates the hypothesis that the disorder is strictly linked to the informational or contextual content conveyed by the sound rather than its perceptual characteristics (such as the loudness or the timbre, for example).

Since Misophonia is a disorder that was ‘discovered’ relatively recently (Jastreboff and Jastreboff, 2002), there have been relatively few epidemiological studies. It might even be wondered whether misophonia is a longstanding disorder which has only recently been assessed, or whether this disorder has on the contrary only emerged recently. We shall return to this point in detail later in this paper when we deal with the history of the civilization of manners. Briefly, we consider the accumulation of social rules and the concomitant increase in sensitivity to transgressions of these rules by others as the causes of misophonia. In this context, misophonia would appear to be a disorder of relatively recent emergence, linked to social conditioning (and potentially other causes). Misophonia would in a way provide a psychological event resulting from a complex ensemble of social ingredients. It is also important to mention that misophonia is generally described for the first time in the course of biological and social development, that is to say during the transition period between the end of childhood and adolescence (Schröder et al., 2013; Swedo et al., 2022).

The prevalence of misophonia varies between studies. It would appear that the prevalence is close to 10% of the general population, although one study reported a prevalence close to 50% (Wu et al., 2014; Naylor et al., 2020; Siepsiak et al., 2020b; Jakubovski et al., 2022; Vitoratou et al., 2023). This variability probably depends on the way misophonia is defined, notably in terms of the severity of the impact on the quality of life. The discomfort related to misophonic sounds (chewing noises, or sniffling, for example) is not all or nothing. These sounds are indeed disagreeable for most people (because we are conditioned to find them abhorrent and to repress them), whereas they are intolerable for misophonic subjects (Enzler et al., 2021b). Among the latter, there is a “leap” in terms of the impact of trigger sounds. The prevalence of misophonia is thus dependent on the level where we place the threshold between disagreeable and intolerable. If this threshold is placed too low, the “false positives” may be very numerous.

## Suggested mechanisms of misophonia

### Misophonia as the result of conditioning

From the early 2000s and the first description of misophonia, Jastreboff proposed a mechanism for the disorder copied from that of tinnitus: *“Finally, the systems and mechanisms outlined above for tinnitus are the same for misophonia, where the tinnitus-related neuronal activity is replaced by activity evoked by external sounds”* (Jastreboff and Jastreboff, 2002). Let us recall here one of the fundamental principles of the Jastreboff model for tinnitus: “*Tinnitus-related neuronal activity acts as a stimulus and the reaction evoked by activation of the sympathetic part of the autonomic nervous system act as reinforcement. Consequently, once the initial association between tinnitus perception and some negative event happening at the same time develops, the reflex loop is rapidly enhanced, as both stimulus and reinforcement are continuously present.*” Briefly, classical conditioning was developed establishing an association between the misophonic stimulus (that is initially neutral) and the affective reaction (anger and disgust). The repetition of the association between the conditioned stimulus and the conditioned response increases the functional connections between the different regions of the brain involved in the processing of information, that is the auditory system and the autonomic and limbic nervous system (Jastreboff and Jastreboff, 2023). According to this model, the negative reinforcement exacerbates any negative emotional or physiological state, and any sound can become misophonic trigger. Following the rules of creating conditioned reflexes, misophonia can be created even if a subject is neither aware of the presence of misophonic trigger nor what is responsible for negative reinforcement. There is no need for any causal link between stimulus and reinforcement, time coincidence is all that is needed, and the conditioned reflex is always created. The initial situation which may trigger the association between the sound stimulus and the reaction is probably very varied and depends on many factors, such as the individual and his personal history, social conditioning and, finally, the way in which the world is expected and perceived. An example has been proposed in the literature: that of a child who is disturbed by the noise from the father’s mouth (Jager et al., 2022). The child has to stay at table and may feel afraid of losing control, so intense are his negative feelings. During the next meal, the child is hyper-vigilant to the eating noises made by the father, he cannot turn his attention away from these noises, which increases still further the discomfort and the negative coupling between the trigger sounds and his reactions. After this classical conditioning, the principles of the operant conditioning maintain and aggravate the symptoms (Jager et al., 2022). We may note that this hypothesis concerning the origins of misophonia suggests clinical treatment based on the extinction of the conditioned reflex. The approach consists in exposing the patients to misophonic sounds while associating them with pleasant situations (Jager et al., 2020, 2022).

It is possible that certain personality traits favor the development of misophonia, such as clinical perfectionism (66–97% of patients) and the imposition of high standards (trait of obsessive-compulsive disorders, 26–52% of patients) (Schröder et al., 2013; Jager et al., 2020). On the other hand, the theory says nothing about the overwhelming prevalence of sounds from the mouth, the throat or the breathing, among the trigger sounds. What is described in the literature tends to suggest that the association between the conditioned stimulus and the conditioned response is accidental or contingent (subject to chance). The unfortunate concomitance of the two events only intensifies the unpleasant aspect of a sound (noise from the mouth or sniffling) which is already disagreeable (Edelstein et al., 2013). It is surprising that so few authors have addressed this nonetheless crucial issue. We shall propose further on an explanation inspired by sociology with the aim of making good this lack.

As we shall see below, the hypothesis developed in this article aims to complete this explanation and also to provide a slightly alternative view. While accepting that the learning and conditioning mechanisms proposed by Jastreboff and others (Jager et al., 2020; Jastreboff and Jastreboff, 2023) play a major role in misophonia, we believe that misophonic sounds are not neutral and that they themselves act as a negative reinforcer. Indeed, we suggest that misophonic sounds have acquired a negative valence as a consequence of social learning and the resulting perceived world. Humans do not look at the world with ‘naked’ eyes, but through glasses shaped by the social environment (Bourdieu, 1979). Their brain is not ‘naked’ in the world (like a *tabula rasa*), waiting to be sculpted by the mechanisms of learning and conditioning at an adult stage of life. He’s already pre-formatted (conditioned) by a social environment that deposits concepts and symbols within him and which structures his mind. The social environment (other human beings) plays a major role, as important as the physical world around him (the trees, plants, animals, landscape, weather etc.). Ultimately, misophonic sounds are perceived as disorder in a world perceived as ordered. A similar hypothesis has been developed for tinnitus: tinnitus (anomaly, turbulence, disorder) is all the more salient the more it is present in the midst of a world perceived as ordered (Norena, 2023). We will see later that the increasing ordering of the world by concepts and symbols is a fundamental trend in human history, fueled by social forces (Elias, 1973; Elias, 2014). Tinnitus and misophonic sounds are sources of disorder as long as they have not been integrated (‘put in order’) into the perceived ordered world. In this context, counseling or psychoeducation, which aims to “demystify” both tinnitus and misophonia, can help to integrate tinnitus and misophonic triggers into the normal, organized perceived world.

### Regions of the brain implicated in misophonia

Certain studies have attempted to identify the regions of the brain activated when misophonic sounds are perceived (Neacsiu et al., 2022). A first study carried out based on IRMf showed that misophonic sounds (presented alone, without context) triggered in misophonic subjects exaggerated cerebral responses in the anterior insular cortex. They were also associated with an abnormal functional connectivity between the anterior insular cortex and the ventromedial prefrontal cortex, the posteromedial cortex, the hippocampus and the amygdala (Kumar et al., 2017). The anterior insular cortex is an important region of the salience network which plays a key role in the perception of interoceptive signals and the related emotional processing (Kumar et al., 2017). Another study where the context was present (the misophonic sounds were associated with the image corresponding to the production of the sound), broadly confirmed the results of the previous study, namely that the anterior insular cortex and the insula were strongly activated (Schröder et al., 2019).

Finally, a recent study proposed the hypothesis that misophonia was linked to the system of mirror neurons. The cerebral activity at rest shows an augmented functional connectivity between the auditory and visual cortex on one hand, and on the other the ventral premotor cortex (involved in orofacial movements). Furthermore, the presentation of the misophonic sounds was linked to a stronger functional connectivity between the auditory cortex and the orofacial motor area. And stronger activation of the orofacial motor area was observed in response to misophonic sounds (Kumar et al., 2021). The authors interpreted these results as evincing an exaggerated representation of the motor execution of misophonic sounds by a third person (a kind of ‘hyper-mirroring’ of the production of the sound). On the basis of this hypothesis, the authors propose clinical treatment targeting the cerebral representation of the movement rather than the representation of the sound (Kumar et al., 2021).

### Misophonia: learning disorders (conditioning) or brain function disorders?

The studies that reveal brain activity in response to misophonic sounds (Kumar et al., 2017; Schröder et al., 2019; Kumar et al., 2021) do not necessarily document the causal chain leading to misophonia. It is not surprising that misophonic sounds activate the regions of the brain involved in the processing of the emotions and vigilance since, by definition, misophonia is associated with intense emotions and hyper-vigilance. We can clearly see the type of circular reasoning the neurosciences can fall prey to: the misophonic sound activates the vigilance network, the subject is misophonic because the misophonic sounds activate the vigilance network. But the activation of the vigilance network may be interpreted as a consequence of the specific valence which the misophonic sounds have acquired one way or another. The question we have is rather the following: how have misophonic sounds acquired a negative valence? In our view, the explanation based on classical conditioning for example proposed by Jastreboff and others is incomplete.

Beyond this question of knowing which areas of the brain are involved in misophonia, we might ask whether misophonia has a functional origin (the disorder is linked to inadequate and/or excessive learning/conditioning, comparable to what happens for phobia, for example) or “structural” (the functioning of the brain is the origin of the disorder, that is the cerebral area is atrophied, this or that anatomical path is deficient – hyperactive, disinhibited etc.). It has been suggested that misophonia may be a form of synesthesia, the cause of which is known in certain cases to be genetic (and thus structural) (Edelstein et al., 2013). Synesthesia is an abnormal association between several senses, where one sense may evoke a sensation in another sense (i.e., numbers, letters or even sounds are associated with colors, for example). Studies have shown that synesthesia was linked to an augmentation of the anatomical connectivity between different regions of the brain. For example, a lesion in the connections between the thalamus and the cortex may give rise to an acquired form of synesthesia where sounds produce tactile sensations (Beauchamp and Ro, 2008). By extension, this hypothesis suggests that misophonia might result from an abnormal anatomical connection between the auditory cortex and the limbic structures. Misophonia would thus be a kind of sound-emotion synesthesia: this or that sound would produce this or that emotion, by a simple triggering of a ‘deviation’ of the neural activity (Edelstein et al., 2013). It is unclear, however, how this hypothesis accounts for the fact that sounds produced by the mouth are most prevalent in misophonia. Indeed, the statistical distribution of misophonic sounds that are bothersome for subjects is not uniform (they often refer to sounds associated to things that are considered disgusting and/or impolite).

## Misophonia: an example of failure in the organization of the perceived world

### The misophonic sounds: disorder in order

One of the striking aspects of misophonia is that the sounds that produce intense discomfort do not appear to be “just any sounds” in everyday life, as has nonetheless been evoked in the literature (Edelstein et al., 2013). On the contrary, it would appear that misophonic sounds belong to a cluster of sounds which can be grouped according to certain common characteristics. These characteristics are fundamental for understanding misophonia. The first characteristic of misophonic sounds is that they are generally produced by humans (some authors, however, report that subjects can be bothered by sounds produced by animals, dog licking itself for example, this point will be addressed later). This property is however not enough to group the misophonic sounds since not all the sounds produced by humans give rise to misophonia. The second characteristic of these sounds is that they are produced during specific situations, often conforming to social norms that are relatively strict and restrictive, which oblige individuals to restrain the production of these sounds. In an online survey where we questioned misophonic subjects regarding trigger sounds, we found that 93% of subjects were bothered by at least one noise from the mouth (chewing, etc.). Some subjects were bothered by other sounds, but they were linked to breathing and the throat. Only one subject (out of a total of 75) was only bothered by repetitive sounds that were not *a priori* linked to the mouth, the nose, the throat or the breathing. Often, the misophonic sounds are therefore associated with behaviors that are under a kind social prohibition, that is to say that should be repressed to avoid bothering anyone, i.e., eating, breathing, noisily clearing the throat. In other words, some misophonic sounds could be related to an attitude that is inappropriate and unacceptable according to the social norms (we will see later that other norms can play a role). Repetitive sounds (repetitive clicking of a pen, for example) may be considered as a kind of generalization of behaviors that should be repressed to avoid bothering anyone. It is also possible that the physical characteristics of the sound may play a role in the discomfort felt by the subjects. We thus felt it was necessary to have recourse to an assessment grid from sociology in order to go further in our understanding of misophonia and its origins. Sociology studies social phenomena, that is the relations, actions and perceptions which emerge dynamically from human interactions. The individual within the society is not only a part of the whole, one of its autonomous elements, s/he is always a society in miniature. We cannot speak of the individual and societies as if we were speaking of a table and a chair, independent and opposable objects. One of the characteristics of human beings is furthermore their extreme plasticity in phase with the social environment in which they develop: they may be a peasant in this or that place and time, or an aristocrat in this or that other context. After a sufficiently early and long period of learning, it would nevertheless be difficult for a peasant to acquire the social codes of the aristocracy, and vice versa (only good actors can achieve this!). The issue here is not to go back in detail over the results and conclusions known and brought up to date by research in sociology (Elias, 1973; Elias, 1975; Bourdieu, 1979), but rather to sketch out the broad traits of the social dynamic that may play a role in a disorder such as misophonia. The social rules and norms are not immutable, they are not ‘floating around’ somewhere waiting to be deposited in the people’s minds. They are on the contrary subject to change, in the course of dynamic processes without any beginning or end, without any purpose, and involving human interdependences at various scales. The social norms are only crystallised and transitory states in these processes which become the focus of the perceptions of sociologists at time t. The general question which interests us here is to know whether there exist social processes which play a role in the onset of misophonia, and if so, to describe them. Furthermore, we wonder whether it might be possible to detect a tendency running through history in the evolution of the social processes which might lead to the present-day emergence of this disorder.

### The civilization of manners

The evolution of social customs in Europe accompanied the pacification of society and in particular the development of courtly society during the Middle Ages. The pacification of manners was achieved progressively at the cost of war and the centralization of power. The lords engaged in what was in fact open rivalry, which resulted in the victors ruling over increasingly vast territories. Society became increasingly complex, with in particular an administration which developed alongside a professional army. The lords were less and less involved in wars, like the other men of the ruling class: a courtly society developed which “invented” social norms. So it was that the word “courtesy” emerged (derived from Old French *cortois*, meaning “belonging to the court”), signifying the codes of refined good manners prevailing at court. Courtesy became a way of behaving within the ruling class but also a mark of identify and of belonging to this class. To be courteous was not only to behave with consideration and politeness toward others, but also to display one’s refinement. There are in courtly society two opposing movements, a centripetal movement of allegiance to the courtly society, and a centrifugal movement of distancing and distinction in relation to the lower social classes. The word “distinguish” (from Latin *“distinguere”*) is synonymous with “separate” and also “discern,” “see” or “recognise.” When we use the expression “to be distinguished,” the meaning of the word shifts to evoke social distinction, that is to say to be elegant, refined, to have good manners. In other words, to be distinguished is to break away from the behavior of the mass of individuals of the lower classes.

In the Middle Ages, the rules governing the good manners of the chivalrous aristocracy were in their infancy and were relatively little constraining. We shall present here a few extracts from the book by Elias (1973), themselves derived from various manuals dictating the rules relative to table manners. We have chosen to focus on table manners because this interests us first and foremost as our main subject is misophonia (and because the sounds of chewing are the most prevalent among the trigger sounds). Furthermore, meals may provoke tension among the participants which is an ideal model for tackling the civilization of manners. A meal is a singular situation which combines a biological need (being nourished and thus mastication, drinking, swallowing, etc. – all these behaviors being visible from others and associated to audible sounds, mouth is also at the interface between the outside and the – disgusting, inside – saliva and various secretions) with social interactions, and thus the more or less imperative duty to be sensitive to the feelings of others. Good table manners thus require self-control, even the acquisition of a certain virtuosity in order to be able to eat both without bothering others and to take part in conversation. To add context to the extracts quoted below, let us recall that the fork did not exist in the Middle Ages and that its use took centuries to become the norm (it was still considered a luxury item in the 17th century). And the handkerchief first appeared in the West after the Middle Ages (Elias, 1973).

*“A few people feel the need to put back on the plate the bones they have finished gnawing; this way of behaving should be rejected, clearing the throat when sitting down at table or blowing the nose on the table cloth are inappropriate things as far as I can judge.”*

*“Drinking from the soup bowl is not proper, even if there are people who are elogious about the cavalier way certain people grab the soup bowl and swallow the contents as if they’d lost their reason.”*

*“Before drinking, you should wipe your mouth so as not to leave any trace of grease on the cup; this rule of courtesy should always be obeyed because it shows courteous intentions.”*

It is noteworthy that making a noise while eating was already in the Middle Ages considered a vulgar and inappropriate attitude:

*“Whoever puffs like a seal when sitting at table, as some people do, or who makes a noise with his mouth like a bumpkin, is likely to be thought of as badly brought up.”*

*“Whoever at table grabs a dish and eats grunting like a pig and making noises with his mouth.”*

*“Whoever puffs like a salmon and clicks his tongue like a badger and clears his throat when sitting down at table is behaving inappropriately.”*

*“For nothing in the world should you noisily slurp up your soup.”*

*“I think it a great incongruity when I see people engaging in the bad habit of drinking like a beast while their mouth is still full of food.”*

The manuals of good behavior thus recommended controlling the ‘animal impulses’ during meals, that is to say behavior that was ‘coarse’ and not ‘polite’ in accordance with the social conventions (avoiding behaving “like a pig,” “like a beast,” “as if he had lost all reason”), and taking care not to inconvenience others (“courteous attitude”). We can see the premises of the psychologization of human relationships in the sense that the behavior also reflects an intention which might offend others. It therefore becomes increasingly important to sense the feelings and expectations of others (Elias, 1975). It is the development of a considerate mode of behavior consisting of avoiding disturbing others. Avoiding making a noise while eating means not only distancing oneself from one’s animal nature, it also means showing consideration and respect to others (of the same social rank), by endeavoring to sense what might upset them. People become gradually more sensitive to the expectations and reactions of others, and the obligation not to wound, scandalize or offend is increasingly pressing. The gradual prohibition of spitting over the course of history is illustrative in terms of the evolution of manners: it was customary to spit on the table during the meal, then under the table when spitting on it was banned, then in a spitoon, and finally spitting was no longer tolerated at all and was banished from acceptable table manners. The same is the case for other customs, such as bodily cleanliness, for example. Keeping the body clean was not initially required for reasons of hygiene but for social reasons: it was appropriate to limit body odor for the sake of others. Perfumes were also used for centuries to mask unpleasant smells.

The struggle for prestige, value and recognition, that is belonging to good society, was fierce. To achieve it, one’s behavior had to be sculpted and shaped (reminiscent of the gardens of Le Nôtre at Versailles), and correspond to the customs of the ruling class. This behavior is also that of complex conventions and protocols, but also of self-control, the internalization of feelings and of discipline. The customs of the upper class became more refined over the course of time to maintain a “distance” (or a distinction) between the upper class and the lower classes. While behaviors initially “crystallised” at court, rather as a crystal forms around an initial “seed,” they then spread to a larger number of elements which in turn crystallized in a centrifugal manner. This spread of the social rules elsewhere than in the restricted circle of the upper class made them seem common and devalued. It thus became essential for the ruling class to innovate permanently with new social rules, to at least maintain absolute rigor concerning the proper execution of the manners of high society. The complexity of manners was such that to master them called for strict and precise conditioning from early childhood on. The training of individuals was so intensive during childhood that it was virtually impossible for the lower class to reproduce the manners of the upper class once a certain critical stage had been passed. And that is exactly the goal of the upper class: to be distinguished from the lower class by limiting the possibility of being imitated.

In the Middle Age, the rules of politeness made their appearance, but they kept an optional character, they had not yet acquired the obligatory and automatic nature they have today. The social pressure which existed with regard to vulgar behaviors was not powerful enough to make them disappear completely. What was perhaps unpleasant but tolerated in the Middle Ages had become indecent and obscene a few centuries later. This is how Norbert Elias distinguishes our epoch from the Middle Ages (Elias, 1973): *“What was lacking in this courtly world [in the Middle Ages] or what did not exist to the same degree as today, was the invisible wall of affective reactions raised between bodies, pushing them away and isolating them, a wall of which we can today feel the presence by a simple gesture of physical rapprochement, a simple contact of an object that has touched the hands or mouth of another person (…)*.”

What changes over the course of history is at once the awareness that we have of the rule and the feeling linked to its violation. Today, the rules are internalized to the point that we are unaware of their origins, they dictate our behavior from behind the scenes in a way that defies our awareness. And yet, taboo behaviors or their simple evocation may trigger in us violent feelings of discomfort, shame, disgust and/or anger. People have striven throughout the “process of civilisation,” as Norbert Elias calls it, to suppress what is interpreted as their animal nature: *“These taboos are nothing other, so far as we can judge, than feelings of displeasure, embarrassment, disgust, anguish or shame, that have been inculcated in humans in specific social circumstances and which have been ritualised and institutionalised* (…).” This animal nature is contained and refined in the same way in their food or their way of eating.

In conclusion, the pressure that people exert on each other has been growing throughout history, paralleled by human behavior that is increasingly controlled, codified and ritualized. Control, distance and discipline are more and more an imperative and the threshold of sensitivity to transgression is in decline: while spitting on the table was considered as inappropriate but finally tolerated in the Middle Ages, it has become repugnant and taboo today. As a behavior or a situation disappears, its emotional resonance increases proportionately, and it becomes increasingly repugnant or vulgar. Society thus changes in a direction where customs become obligations, where certain behaviors are eliminated, and where the sensitivity to a behavior assumed to have disappeared increases. This is one of the main lessons of this socio-historical reflection: the pacification of manners and the normalization of behaviors comes at the cost of an increase in sensitivity, which itself demands still more pacification and more rules. Society is in a blind and dynamic process of regulation and control. To illustrate this point, we may evoke the slaughtering and butchering of animals, a practice that was common in the Middle Ages, and in the countryside not so long ago. The disappearance of these practices from the public space has transformed the sensitivity of individuals to the point where it would be intolerable for many people today to watch this slaughtering, *a fortiori* all the more when it concerns young animals which arouse pity, i.e., lambs and suckling pigs. And yet, many people enjoy eating the flesh of these animals. This tendency continues today even in research laboratories, with the development of “animal wellbeing” and the spread of lobbying to stop the use of animals for research purposes, using photos that shock, for example.

Humans have replaced spontaneous, “natural” behavior with intelligible social symbols. In the same way, humans have mapped “nature” (let us say the non-human world), and even his relationship with time (Elias, 2014). This symbolic shaping of his environment (human and non-human), i.e., their synthesis by abstract concepts, functions for humans as an instrument of orientation in the world. It allows humans to constantly extend their control over his environment (human and non-human), thereby lowering the level of danger to which they are exposed. We believe that the strong emotional reactions associated with any turbulence or disorder in the organized world as it is perceived, is linked to this need to control the environment in order to reduce uncertainty and danger. Positive emotions are associated with a feeling of power (success in survival), while negative emotions are associated with a feeling of powerlessness (failure in a survival situation). Anger, for example, is the emotion that can follow disappointment, confronting it and refusing to leave it at that. Finally, as we shall see later, the price to be paid for a world that is perceived as increasingly symbolic and organized is heightened sensitivity to the slightest factors of disorder.

### Misophonia: turbulence in the perceived world

We now come to the question of knowing how the social processes may play a role in the emergence of misophonia. We have seen with Norbert Elias that the pacification of relations between people, linked to their interdependence, imposed an ever-increasing number of social rules, among which the courtesy of the court was the laboratory. Whereas certain rules consist in hiding certain situations (nudity, sexual relations, violence, slaughtering animals, etc.), others codified social situations, for example table manners. These rules which were initially simple precepts (advice on good behavior) in time became injunctions, or even taboos, associated with a huge affective dimension. After the generalization of the fork and the handkerchief, eating with the hands, spitting on the table, blowing one’s nose in one’s clothing, clearing the throat, became forbidden behaviors, strictly controlled, and with a strong emotional connotation. The suppression of these behaviors was achieved at the cost of a strong association between the actions to be repressed and the whole repertoire of negative feelings (fear, shame, disgust). It is striking to note that our own saliva does not produce disgust as long as it is in our mouth, and that it become repugnant if it is spat out into our hand! We note that the mouth is the visible boundary between the outside and the – disgusting – inside of the body (saliva, secretions from the nose, pre-digestion of food, bad smell etc.). It is therefore not surprising to find sounds from the mouth (chewing, swallowing, clearing the throat etc.) among the most prevalent misophonic sound triggers. Of course, the sounds or behaviors that become taboo depend very much on the culture of the society. In Japan or China, for example, it’s not impolite to eat noodle soup while making a lot of noise (whereas it is in France). Conversely, it is impolite to wear perfume in Japan (whereas it is not the case in France) because it is interpreted an individualist behavior. Spitting in the street is widely regarded as very impolite and disgusting in France, whereas it is broadly tolerated in China. The collateral damage of the pacified society of today, regulated and individualistic, is the permanence of the social prohibition: toward oneself, that is reflected in the fear and anguish of breaking the rules, and toward others by heightened sensitivity toward their behavior (toward the possibility that they may transgress the rules).

Our hypothesis is that the origins of misophonia are to be found in this social conditioning, with the addition of an exaggeration: misophonia may be seen as a heightened sensitivity to the failings of others with regard to their obligations of good behavior and considerateness, and taking food is just one example among many. In other words, social rules organize and structure the perceived world and are therefore a possible origin of human’s sensitivity to factors that add disorder to his perceived world (which are not integrated into the perceived world). The misophonic sound does not only trigger simple disapproval vis-à-vis the breach of the rules, but on the contrary a strong feeling of disgust, indignation, anger, even protestation. What is at play in the misophonic subject is the intimate way they experience the breach of the rules. The breach of the rules must be interpreted in three different, but not clearly separate and largely overlapping contexts.

#### Sociability failure

In the first situation, the subject is open to others, respects social rules and expects the same from others. However, the violation of rules by others can be interpreted as a disrespectful and asocial intention, thus closing off the possibility of a social relationship. The other is judged as not being ready to play the social game with a view to establishing a relationship based on the beginnings of trust, i.e., not alienating oneself in pleasure and the object, keeping a distance, and always considering the other as an end (a person and not an object, whose respect takes priority). It’s not simply a question of violating a rule of decorum, because the rule of decorum has become a moral rule (politeness, respect, trust, reliability, etc.), and it’s a moral rule that is being transgressed, which is much more serious. As soon as a social rule is transgressed, a fortiori when it has been invested with a moral value, this can trigger strong reactions of contestation. Even a sound as neutral as footsteps might be for most people can become bothersome and trigger strong reactions. In fact, footsteps were a misophonic trigger for a patient whose stepmother, with whom relations were not good, spied on him and discreetly followed him around the house. This example, which was brought to my attention by a colleague, illustrates how a sound can acquire a negative value and can generalize to any sound of the same category (here the category of “footsteps”). In a second stage, the sound and its acoustic characteristics become a kind of imprint of the disturbing situation that produced the conditioning. Importantly, the negative value that has been associated to the sound depends strongly on a broad and meaningful context for the patient. The act of spying is morally reprehensible for several reasons: one person violates the privacy of another without their knowledge, it makes the relationship between the protagonists asymmetrical and, finally, the intention behind the act of spying may also be reprehensible (the spy may use the information for their own interests, which may not coincide with those of the person being spied on). This situation is also deeply intrusive and disturbing, because the act of spying is by definition hidden, and the stress and vigilance associated with it are both prolonged and increased by the ever-present possibility of the spy being around. Finally, the situation may also be perceived as even more violent since the person spying is not the patient’s mother but the stepmother (who may not have the legitimacy to do so in patient’s mind). Here, we hypothesize that it is the violation/transgression of a moral rule by the stepmother that is at the root of the patient’s initial reactions, and that the reactions are so strong that the association between the trigger and the reactions is generalized to other contexts. This view is reminiscent of the theory of Attribution Process (Jones and Davis, 1965), which aims to account for the ability to apprehend the personal dispositions (intentions) of individuals through their behavior. It also evokes the thinking of Kant, for whom reflection on morality should not start from the Good, but from the moral requirement, i.e., from the subject itself, his intention endowed with freedom and reason. This moral requirement takes the form of three “categorical imperatives”: “*Act in such a way that the maxim of your action can be set up by your will as a universal law; act in such a way that you always treat humanity in yourself and in others as an end and never as a means; act as if you were both legislator and subject in the republic of free and reasonable wills*” (Kant, 1993). Kant is not suggesting that we must follow the rule as such without thinking; he is emphasizing intention and respect for the rule, so that it is the approach (generalizable to the whole of humanity) that ultimately constitutes the law.

#### Disgust for other people’s taste

In a second situation, the subject is no longer in an attitude of openness, but of closure toward others. What’s disturbing is not that the subject is violating a moral rule in relation to others, but that he or she is violating an esthetic rule, i.e., a rule of taste (although esthetics can be considered a moral). This is the social game that marks membership of the dominant class. The accumulation of value or prestige is inseparable from recognition, one being derived from the other. As Pierre Bourdieu sums up in his book La Distinction (Bourdieu, 1979): “*Tastes are above all disgusts, made up of visceral horror or intolerance of other people’s tastes*.” Indeed, “*This horror, ignored by those who abandon themselves to sensation, results, fundamentally, from the abolition of the distance, where freedom asserts itself, between the representation and the thing represented, in short from the alienation of the loss of the subject in the object, from the immediate submission to the immediate present determined by the enslaving violence of the “pleasant.”* Taste, in art as in lifestyle, is a powerful social construct: it must distinguish itself from the easy, the accessible, the primitive, the rakish – in short, from the vulgar. Thus, the principle of pure taste is above all a refusal! “*The world that produces artistic “creation” is not just “another nature” but a “counter-nature,” a world produced in the manner of nature but against the ordinary laws of nature*.” It is possible to establish a link between the Good (morality) and the Beautiful (esthetics). Where there is control and distancing from immediate pleasures that flatter the body and senses (food intake), behavior then appears distinguished. In the case of anorexia (see below), Beauty and Goodness are intertwined, with the ideal of a beautiful body (sculpted and controlled) in tune with morality (refusal of immediate pleasure, alienation of thought from sensual pleasures). This idea refers back to Bourdieu’s analysis of the Beautiful, which he links not to a universal, but more surely to a complex social game implicit and internalized by the players: “*In such a way that the ‘purest’ form of the aesthete’s pleasure, the purified, sublimated, denied aisthesis, could thus constitute, paradoxically, in an asceticism, askesis, a tension trained and maintained, which is the very opposite of the primary and primitive aesthesis*” (Bourdieu, 1979). To return to misophonia, the subject who makes noise while eating indulges in a primal, alienating pleasure, a vulgar and debonair attitude, in contradiction with the “laws” of good manners that impose restraint, distance, the internalization of pleasure, and above all its control.

An example of lifestyle conflict and how a distance exists between people of different social classes is the problem of noise pollution complained of by city dwellers who move to the countryside. These people have the purchasing power to move to the countryside after selling their property in the city. Living in the countryside is a dream come true: a bigger house, access to a vast outdoor space, a healthier, more serene lifestyle and fewer nuisances, especially noise. However, many of those who move to the countryside are disappointed, as they had imagined an idealized countryside (a sort of sanitized countryside, a “green Disneyland”) that ultimately does not correspond to the perceived world they had imagined. In reality, the “real” countryside can be noisy, as it is populated by domestic animals that make noise (cowbells) and vocalizations (roosters, geese, dogs, etc.), not to mention the smell of their excrement! In France, the number of such complaints has exploded with the increase in the number of city dwellers moving to the countryside (particularly after the COVID containment episode, which played a role in accelerating this “return to the countryside”). Whereas city dwellers were bothered by urban noises but tolerated them because they were part of the world they perceived, it is very difficult for them to tolerate animal noises in the countryside. We assume that these noises are intolerable because they are linked to human activity. Yet this human activity is discredited by many city dwellers, who see it only as a boorish, vulgar way of life. For city dwellers, the esthetic or moral fault lies with those who have always lived in the countryside, and who must adapt to the more refined, sophisticated – in a word, superior – lifestyle of city dwellers.

#### The order-disorder balance

The third situation is one where humans have no *a priori* responsibility for the nuisance. A caricature of this situation was recently described in Provence (France), where French and foreign tourists complained that they could not stand the song of the cicadas. It’s true that cicadas can be very loud (up to 90 dB). But for the locals, this song is part of the perceived world, perfectly integrated into the background noise, like the blue of the sky, always present and at the same time “transparent” insofar as it is not particularly prominent in relation to the other sensory elements of the perceived world. The song of the cicadas is part of Provençal identity in summer, the season of vacations, holidays, balmy nights and sociability, which even gives these songs a positive connotation for the inhabitants. However, the emotional connotation is negative for some tourists. More generally, it is possible that misophonic subjects who report discomfort caused by objects (fans, refrigerators, printers, airplanes, etc.) result from the same cause as that at work for cicada singing. To describe this putative mechanism, we need to look briefly at the way in which human consciousness unfolds in the world (Norena, 2023).

For Schopenhauer (1818), the world can be seen as a human will. The world as we experience it is first and foremost the product of our openness to it, of our desires, and therefore of our will. Nietzsche says as much in this passage (Nietzsche, 2000): “*Assuredly they have dared more, innovated more, braved more, provoked destiny more than all the other animals together: they, the great experimenters who experiment on themselves, dissatisfied, insatiable, who battle for supreme power against the animal, nature and the gods–they, still untamed, the beings of the eternal future who finds no repose from their strength, pushed unceasingly by the burning spur that the future plunges into the flesh of the present: they, the bravest animal, the one with the richest blood, how could they not be exposed to the longest lasting and the most terrible of the diseases among those that afflict the animal?*.” In addition, human consciousness always aims at a content, an intentional object (real or imagined) that it is always in tension with the world. This characteristic of consciousness defines what Husserl calls its “intentionality,” that is, the fact that it is the bearer of an intention, that it is open and directed toward something other than itself: “*any state of consciousness in general is, in itself, consciousness of something*” (Husserl, 1931). The important point is that this mode of consciousness is spontaneous, unconscious and inevitable (unless you practice Buddhist meditation or Husserl’s phenomenology).

Merleau-Ponty, a disciple of Husserl, asserts that Man merges with the world in the sense that he is in permanent resonance with it, that he forms a system with it, to such an extent that it would be pointless to establish a distinction between the subject and the world (Merleau-Ponty, 1985, 2009). In other words, the subject is not separated from the world, he participates ontologically in the things he’s perceiving. “*Nature is within*,” said Cezanne (famous French painter). “*Quality, light, colour, depth, which are over there in front of us, are only there because they awaken an echo in our own body, because it welcomes them*” (Merleau-Ponty, 2009). The drawing and the painting are not a copy of the world, they are “*the inside of the outside and the outside of the inside*” (Merleau-Ponty, 2009). In Le Petit Prince (Saint-Exupery, 2013), the pilot of the plane that broke down in the desert spoke to the desert landscape: “*I’ve always loved the desert. One sits on a sand dune. One sees nothing. One hears nothing. And yet something radiates in silence* (…).” This passage illustrates what Merleau-Ponty describes: the world (landscape) and its components (rocks, trees etc.) seem to vibrate or radiate energy and life, and they are animated by our tension and connection with the world, the intentionality of consciousness. The world is animated by man’s intention and rules: this is how the world can be “divine” (i.e., sacred, spiritual, pure, beautiful, well-ordered, shaped, balanced etc.) or “turbulent” (i.e., shapeless, monstruous, awful, repulsive, excessive, etc.). A desert can be full of life and beauty for a given person, whereas it can be an empty and boring place for another (same for mountains, oceans, cities, forests etc.).

The ways In which we connect intimately with the world are infinite and their detailed mechanisms are probably very complex and subject to change via learning and conditioning. In this context, the sound of rain can be considered relaxing as long as it is perceived as harmless, and from a comfortable and soothing situation, but this association can change if rain is associated with flooding and the damage it causes. In this latter case, rain can be a source of anxiety. Moreover, while a sunset can be breathtaking, a wind farm can be ugly and the sound of a barking dog can be irritating. The world we perceive and live in is organized according to the social rules we have learned and the rules we ourselves have developed over the course of our lives. In this context, the dog’s barking may be annoying because it breaks the silence and/or because the dog should be trained not to bark. A barking dog can therefore violate an esthetic rule (silence is beautiful, or allows to hear birds singing, etc.) and/or a moral rule (the owner should have trained his animal correctly to respect the peace and quiet of others).

Beside the order–disorder (sensibility to order and/or ability to integrate a disorder into another order), any (neutral) sound can become a trigger sound, as a result of classical conditioning (Jastreboff and Jastreboff, 2023). In this case, a fortuitous association between a stimulus and an emotion can durably, perhaps definitively, link the stimulus to the emotion (see the Little Albert experiment with white mice), whereas the two are strictly independent.

#### Summary

The sound that becomes a misophonic trigger may be unpleasant from the outset for social reasons (mouth noise, throat clearing, etc.), and its unpleasantness may be increased (to the point of becoming problematic for the course of life) by additional conditioning, namely repeated exposure to this sound, in an unpleasant context (possibly social: tense family gatherings, high expectations on the part of loved ones, education, personality traits etc.). Other sounds, on the other hand, are not immediately unpleasant, but become so when associated with a particular negative context (the footsteps of the child’s stepmother). The negative reinforcer leading to the stimulus-emotion association is not independent of the stimulus but, on the contrary, is closely linked to it. Finally, any sound can become a misophonic trigger due to classical conditioning. The acoustic characteristics of the sounds could also play a role, as some of them (energy in the 2,500 Hz–5,500 Hz region, modulation frequency around 40 Hz, inharmonicity etc.) seem to make the sounds more unpleasant (Kumar et al., 2008; Arnal et al., 2019).

In addition, any sound may take a negative connotation if it is not harmoniously integrated into the perceived world. The sound is then perceived as turbulence or disorder, in a perceived world that is intended to be orderly. The important point is that the very structure of consciousness, i.e., its will, its openness to the world, which it tirelessly seeks to link together and form a coherent perceived world (notably through the social symbols that organize it), independently of any concomitant negative event, can confer a negative valence on a stimulus. This last aspect probably plays a role in all the scenarios mentioned above. Moreover, the perceived world is all the more sensitive to disorder when it is strictly ordered, i.e., organized into concepts and symbols of a high level of synthesis. As already mentioned, the driving force structuring the perceived world (and thought) is social. The meaning that is given to the turbulent element is also important in giving it its character of anomaly and disorder, and this meaning is eminently social. It is also possible that this mechanism plays a role in the acceptance or non-acceptance of tinnitus, i.e., in its integration into the world perceived as normal and non-turbulent. Beyond its psychophysical properties (loudness, for example), tinnitus is perceived as a thing or object that disturbs the harmony of the perceived world by adding an element of disorder. Tinnitus can thus give rise to both discomfort and hypervigilance, depending on the subject’s sensitivity to this disorder and his or her ability to integrate this background noise into his or her perceived world (Norena, 2023). Misophonic sound and tinnitus “pop-out” from the coherent and ordered auditory scene as they are perceived as a disorder.

To return to Kant, beyond the explanatory power of his thought, we are also interested in his thought insofar as it illustrates an approach or structure of thought that can be generalized to the whole of humanity. In other words, it is possible that Kant formulates in a concrete and rigorous way a thought of moral attitude that other people are capable, if not of expressing, at least of thinking more or less explicitly like Kant, at the same level of moral requirement as Kant (taking into account all the categorical imperatives) or at a reduced and less strict level. Kant’s thought would therefore say something not only about morality (a criticizable reflection), but also about a common way of understanding and practicing it (a structure of thought that is an observable fact in all philosophical works). Philosophy, insofar as it always starts from a thinking subject who strives to think the true and the universal or the generalizable, can be seen as an original approach to understanding the human mind. Every philosophical systems or thoughts, every prejudices, can be seen as motifs of human thought, the concretizations of reflections and meditations arising from the unconscious foundations of consciousness. Insofar as these thoughts and systems are accessible facts in the works of philosophers, a psychology is possible based on a history of philosophy. This original attitude to philosophy, both critical and constructive, is developed throughout Nietzsche’s work (Wotling, 1999; Nietzsche, 2022a). “*Who then, before me, has descended into the caverns from which springs the poisonous breath of this species of ideal, the ideal of the world’s slanderers? Who then dared to suspect that these were caves? Who then, before me, was, among philosophers, a psychologist, and not the opposite of a psychologist, a “superior charlatan,” an “idealist”? Before me, there was no psychology*” (Nietzsche, 2023). For Nietzsche, there is no such thing as a “pure mind,” free and autonomous, capable of leading to truth. For him, in reality there is no truth, or rather, truth is not an essence but a value emanating from the unconscious mechanisms of thought. Philosophical thought only responds to a need for self-preservation, enabling life to continue. It’s the body that thinks, and thought is an epiphenomenon. In this context, we speculate that emotions can be seen as the body’s messages to consciousness, a kind of degradation of consciousness initiated by the body, in the form of charm and dream, for the purpose of survival (Sartre, 2000; Norena, 2023): successes are associated with joy and a sense of power, while failures are associated with sadness, anger, frustration and a feeling of powerlessness. In short, for Nietzsche, the task of philosophy is a psychology, it is to unearth the buried structures of thought behind the “visible” or in other words the symptoms: “It is legitimate to consider the audacious follies of metaphysics, and particularly the answers it gives to the question of the value of existence, first of all as symptoms of bodily constitutions peculiar to certain individuals (…)” (Nietzsche, 2020). Nietzsche’s genealogical approach is summed up in this passage: “In truth, you cannot be a philologist and a doctor without also being an ‘antichrist’. The philologist looks behind the Holy Books, the physician behind the physiological degradation of the typical Christian. The doctor says: “incurable,” the philologist: “imposture” (Nietzsche, 2022b).”

One wonders whether Kant, a great philosopher of Morality, was suffering from misophonia, or a form of extreme inflexibility with regard to compliance with the rule! For Kant (and the misophonic subject), it’s possible that respect for the rule goes beyond the local and, at first sight, minor, derisory, inconsequential character of a given situation. They show great sensitivity and/or lucidity with regard to the consequences, however remote (in time and space), and without the possibility of calming their anxiety, of other people’s actions, especially those that violate a moral rule: if I lie, others may lie; if everyone lies, no one can be trusted; if the establishment of a relationship of trust is not possible between men, no society can be envisaged other than in terms of power relations. For Kant, this concern for patience and restraint is the quality that differentiates humans from animals, the quality that ultimately makes society and living together possible: “*This power not only to enjoy the present moment of life, but to represent in a present way an often very distant future, is the most distinctive sign of man’s superiority*” (Kant, 2008). Kant’s biographies and his epistolary correspondence provide an insight into his life and personality. It is reported that the great philosopher had deep-seated anxieties, as well as numerous quirks and obsessions. For example, he almost never deviated from a strictly regulated daily routine, from waking up in the morning to going to bed at night. Living near the Pregel River, he complained of the unpleasant sounds of boats and Polish carts. From one of his lodgings, Kant also complained of hearing singing from the neighboring prison! On another occasion, he asked a neighbor to cut down his trees because they were blocking his view of the city’s tallest tower! But what bothered him most in his work and thinking was the crowing of a rooster! Kant tried to buy the animal he found unbearable in order to find peace and silence, but having failed in his project he finally decided to move! It seems that Kant had an exacerbated sensitivity to order, and little facility in integrating disorder, particularly but not only with regard to sound sources.

### Misophonia and other factors

#### Personality traits and/or social environment, education?

The explanation of misophonia developed above does not fully exhaust the possible explanations of misophonic disorder, but it provides an assessment framework which we feel is pertinent to at least partly understand its origins. Nevertheless, if we are all to some extent conditioned by the same “forces,” not everyone complains about misophonic sounds with the same intensity. Is the problem linked to the presence of certain personality traits which might be present in misophonic subjects? The literature reports that a majority of subjects (97%) present clinical perfectionism (Shafran et al., 2002; Jager et al., 2020). Or is it a matter of conditioning (education) and the social context in which it was forged? Misophonic disorder develops on the basis of exposure to a core of situations which expose the subject to a negative feeling that is repeated with no possibility of escape. It is very possible that this core is to be found in the subject’s immediate environment, that is in the close family circle (parents, brothers and sisters), inasmuch as the family is the initial place of conditioning. The subjects report besides that the discomfort is all the more severe when the sounds are produced by close relations (Jager et al., 2020). Family environment is the place of initial conditioning (see below the section on childhood and adolescence). The problematic situation is thus progressively feared, fear and anxiety develop, aggravating the disorder with each exposure. The subject’s apprehension is accompanied by hyper-vigilance which amplifies the discomfort. A vicious circle develops to which are added avoidance behaviors. The conditioning then becomes generalized to all trigger sounds. One may note that the conditioning is such that the simple presentation of misophonic sounds without context produces a strong reaction in the misophonic subject (Kumar et al., 2017; Enzler et al., 2021b).

#### Social conditioning: from childhood to adolescence

Social customs, what is allowed, encouraged or forbidden, what is polite or vulgar, what is brilliant or mediocre (Bourdieu, 1979), are fixed throughout a child’s development up until adulthood. This is social conditioning, exercised from outside, first by the parents, then by the immediate family, then by the network of more distant relatives (the whole society: friends, school, sports club, etc.), throughout which the child will be taught to eat with their mouth closed, not to talk with their mouth full, to wash their hands, not to put their fingers in their mouth, their nose, etc. It is important to stress that vulgar and inappropriate behaviors are eliminated by the classical conditioning and the use of negative reinforcement, i.e., which are the negative emotions such as shame, disgust, fear and perhaps also anger: “You must not do that, it’s dirty and disgusting!,” “Are not you ashamed to make so much noise when you are eating?,” “If you do not blow you nose straight away, you’ll be punished,” “It’s infuriating to see the person next to your at table showing such bad manners when they are eating,” etc. The forbidden behaviors are thus from the outset associated with disagreeable or even violent affects, which are related to guilt, terror and disgust. We can see clearly that if on one hand the conditioning should lead to the stabilization and the internalization of the prohibition, it should at the same time integrate a certain flexibility or tolerance on pain of turning out to being too rigid and counter-productive. The social conditioning should not be done at the cost of excessive rigorousness and intransigence.

During development, children should very quickly integrate the level of prudishness, sensitivity and politeness corresponding to their social class. Norbert Elias commented that the development of the individual recapitulates the history of the civilization of manners (Elias, 1975): *“The history of a society is reflected in the internal history of each individual: each individual must on their own account go through a condensed form of the process of civilisation that the society has gone though in its entirety; because a child is not born civilised.”* Furthermore, as the rules of the society increase and become more complex, the critical period of social maturation is longer and longer (Elias, 1975): *“The gradual transformation of the individual as they grow up, the individual process of civilisation through which, starting from the universally identical base of a child’s behaviour, they come closer and closer to the level of civilisation achieved by the society, becomes increasingly difficult, it takes longer and longer: the time needed to prepare the child who is growing up for the roles and responsibilities of an adult, gets longer.”* A child or an adolescent who does not achieve the affective norm required by their social environment, in whom the conditioning has partly failed, will be considered as “abnormal.” Adolescence is a crucial stage in development in terms of the internalization of the rule. Whereas a child is content to obey the injunctions of their educators without fully understanding the social issues at stake and the motivations of their elders, in adolescents emotion has definitively taken root, with intensities that may be particularly high, which is the reflection of a condition that is raw and recent, not yet “polished” by a few more years of social life. The adolescent tends to be excessively strict.

In everything we have described above, the notion of distance between people is of capital importance. It is not just a matter of protecting others from what our body and our mind may produce that is vulgar or disagreeable, but also that others may impose on themselves the same rules that we endeavor to apply to ourselves. In other words, these manners define the right distance that must be observed between individuals: it should not be too close, so as not to intrude on others, nor too distant so as to allow exchanges and show respect for one’s interlocutor. We speak of “keeping your distance,” an expression that reflects a defensive and closed position, a sort of bastion or fortress that must not be invaded. This bastion is the private space which is strongly dependent on the society and the individual (Sorokowska et al., 2017). Conversely, we use the expression “to make yourself at home” (or “to stay cool,” “to hang loose”) to describe an open behavior which is deployed without social restriction and with confidence outside the subject, through their behavior, i.e., gestures, words, attitudes, clothes, makeup, hairstyle, etc. It goes without saying that these distances are highly variable and dependent on a whole series of laws (most of them unconscious) and on the context (sitting round a table for a meal, degrees of familiarity, hierarchical relations, etc.). But adolescence is the period when these distances are fixed and when the sensitivity to intrusion is exacerbated. Today, music is ever-present in an adolescent’s life and plays an important social role. It serves as an outlet for repressed emotions. Furthermore, if the bedroom is the adolescent’s private space customized to their taste with their own decorations, music creates the adolescent’s own sound space, it screens (masks) the sounds from outside. The two contribute to creating a “refuge” providing protection against intrusion by establishing a certain distance from the outside world.

Another major consequence of the social rules which have finally been imposed is the notion of interiority. Once it becomes vital to control one’s acts, to disguise or mask one’s real impulses, a space is created within the individual: *“And we may consider as characteristic of a certain phase of this process [of civilisation] the accentuation of the tensions between the constraints that the individual imposes on themselves under the effect of the social commandments and prohibitions and the spontaneous impulses which they repress. It is (…) this contradiction within the individual, this “interiorisation,” the fact that certain spheres of life are excluded from social intercourse and hedged in by feelings of anguish, shame and embarrassment of social origin, which gives rise in the individual to the impression of being “inside” something for them alone, which only exists for the others, “outside,” a posteriori”* (Elias, 1975). This “inner self” is the ultimate refuge, that enables us to have a private and defended existence sheltered from the eyes of others. Yet the important conclusion for our subject is that the feeling of intrusion or aggression is defined by the inner-self or interiority (i.e., that which is not shared with others, that which is distant from others), and that this feeling is all the stronger the more developed the private sphere. In other words, the feeling of intrusion is a direct consequence linked to the separation of individuals, to the individualization of society and to the inner self.

The misophonic subject experiences the trigger sound as an intrusion and an assault on their “inner self,” that is to say as an act of violence with regard to the relation with the pacified and regulated world that they expect. The inner self, where the affects are private, is a consequence of the internal tension between the social obligations and the subject’s instinctive tendencies. The essential condition for a feeling of assault to develop is the existence of an inner self. We may note that the importance of the inner self and the associated sensitivity to assault may play a role in other disorders, such as tinnitus or hyperacusis, for example (Norena, 2023). The assault experienced is all the more serious inasmuch as the inner self is important, and thus as the inner tension within the individual is strong. This tension probably reflects the level of compliance of the subject toward the rule and/or the behavior or thought which is the cause. The problem of the misophonic subject is not the sound itself, it is the other’s whole cognitive process, from their thoughts to their acts, which cause the sound: someone who is a noisy eater, who sprawls around, who behaves too casually, who does not keep their distance, and who encroaches on the inner private space of the misophonic subject. This encroachment of the other is experienced as an “unlawful” invasion, arousing anger and aggression.

#### Intention

Misophonic subjects well know that misophonic sounds are not produced intentionally by those who emit them (Edelstein et al., 2013). But the problem goes beyond the conscious intention: thoughtless sloppiness, laziness, negligence, an inconsiderate attitude, a lack of rigor in self-control, are just as unacceptable as a deliberate transgression. The behavior is judged as insufficiently precise and refined, the self-discipline required to respect the distance from the other is lacking or inadequate, and that is intolerable. The misophonic disorder is related to the moral obligations of others, their duty to show restraint within the social space. In this context, the sounds which are normally triggers when they are produced by an adult are not triggers when they are produced by a child (Jager et al., 2020). The adult of an age to understand the social rules is held responsible for their acts: they transgress a prohibition; they are guilty of a lack of self-control. The child like a subject with a mental disorder or an elderly person with dementia, are, on the contrary, considered guiltless, in other words they are innocent in their attitudes. This last aspect is certainly variable from one misophonic subject to another and depends on the way the conditioning was developed and on the context.

#### The sense of hearing

Misophonia is a disorder that is relatively prevalent since it is linked to sound, and sound reduces the distances by overcoming the physical [*“The ear has no eyelids,”* (Quignard, 1997)]. Intrusive noise, which may give rise to violence (Dzhambov and Dimitrova, 2014), exists even and above all when one is at home or in one’s bedroom. If the reaction of the misophonic subject is extreme, it is because the sound cannot be avoided or ignored (Norena, 2023). One might imagine that visual situations may produce disagreeable reactions (Schröder et al., 2013), in the same vein as those produced by sounds. It is for example possible to feel contempt or even disgust at certain attitudes. One might be upset by a lack of elegance regarding a certain posture, for example. One might contrast the maintenance of the body, its stiffness or even rigidity (women’s corsets since the Middle Ages), the sign of a strict attachment to the social codes in force and self-discipline, with the sloppy attitude, casual and less appropriate, of the lower classes. The head that nods in time to the music at the opera is also interpreted as a behavior that may be seen as a breach of the codes of polite society (Degiovanni, 2023). This behavior, which may seem harmless to those who are uninitiated in the codes of the opera, is not a simple external sign of naivety. In reality it casts aspersions on the person by discrediting them. They are suspected of not understanding or interiorizing the music, “of overdoing it,” in other words of being out of place in the milieu of the upper or educated classes who know how to appreciate art. The right attitude is above all not to give way to the emotions and keep one’s self-control. Refinement requires sang-froid and control. In this sense, the opera whose codes (based on restraint) date back to the courtly epoch contrasts with contemporary popular music (rock, pop etc.) based on expressing the emotions (Bourdieu, 1979). It is however rare that these types of visual situations engender as severe a discomfort as those produced by sound; perhaps the visual events do not provoke as sharp, or intrusive, sensation as those associated with sound stimulation. The specificities of the auditory thus play an important role in misophonic disorder, similarly to what occurs for tinnitus and hyperacusis (Noreña et al., 2021).

### The balance between ‘I’ and ‘We’

Once these associations have been learned, the rules are internalized and conserved by an unconscious process of self-control. We may note to what extent the social structures are anchored in the deepest recesses of our minds and of our emotions (shame, embarrassment, disgust, anger). In passing, this is a critique of psychology and Freudian psychoanalysis (Elias, 2010) which does not take enough into account the social factors that shape our behavior: *“The concept inscribed at the base of all these theses is the opposition between a “pure self” – the subject matter of psychology – which would in some way enter post hoc in contact with others and with a society – the subject matter of sociology – facing the individual as an entity existing outside himself.”* The concepts of “ego” and “super-ego” are not only individual adaptations of the subject to the social world, adaptations which differentiate individuals from others. On the contrary, the “ego” and “super-ego,” are profoundly marked by social structures common to all individuals: *“the psychological and psychiatric theories exclusively centred on the perspective of the “I” […] suffer from an exuberance of the function ‘I’ in the balance between the levels of “I” and “we”* (Elias, 2010). In this sense too, there would not be a “constant human nature,” as described by Freud through the “libido,” “the instinct of aggressiveness” or the “death wish.” When there is no point zero where human beings would have lived in the natural state (independently of society/culture), it would be sterile to oppose nature and culture. The nature/culture opposition has no more basis than the opposition individual/society (Elias, 2010).

### Digression on anorexia

Anorexia (or eating disorders) is a form of asceticism, that is a chosen discipline of the body and/ or the mind with the aim of attempting to arrive at an ideal form of achievement. Nietzche underlined the ancient and vastly ambitious nature of asceticism: *“This should be a necessity of a higher order which makes grow and prosper this species hostile to life — life itself should have an interest in not allowing to perish this contradictory type. For an ascetic life is a flagrant contradiction: a resentment without purpose dominates, that of an instinct which is not satisfied, a desire for power which would be master, not of something in life, but of life itself, of its most profound, strongest, most fundamental conditions, (…)”* (Nietzsche, 2000).

We feel that the assessment framework developed above to at least partly explain the origins of misophonia may also be applied to anorexia. Society today presents a huge paradox. On one hand, the freedom to dispose of opportunities, allowing the possibility of choosing one’s way of life, is greater than in the past. Social determinism is still present, but the individual can develop in a broader space of possibilities, at least theoretically. A farmer’s son can go to a prestigious university and climb the social ladder. This freedom of destiny comes however at a high cost. The fact of taking control of one’s destiny is the source of a two-fold anguish, that of failure which cannot be imputed to factors outside the self, and that of not making the right choice of lifestyle or career among the vast range of choices that are offered to every individual (Elias, 1991; Ehrenberg, 2001; Ehrenberger, 2018). The freedom of choice is terrifying! In this context, one may wish to escape from the shame of failure by success and monumental achievement. Furthermore, value should be won thanks to one’s intrinsic qualities, autonomy, work and discipline. The figure of the autodidact, who achieves success alone, without ever giving up in the face of adversity, is a classic case of success today. On the other hand, the road to success is hedged by the very strict limits the individual must respect on pain of arousing suspicion and contempt (Elias, 1991). The individual must indeed be in phase with their social rank and observe strict control of their affects and their attitudes, as outlined above. We can see how difficult it may be today to make a success of one’s life, and the pressure that weighs on young people in the course of their development, waiting to be integrated into society.

Anorexia may be a response to this double injunction of success and self-control (Merand and Lemoine, 2022). Young girls “choose” the alternative of anorexia to try to match an ideal which is expected of them. They embark on this “adventure” as one might decide to go around the world or go on pilgrimage to Santiago de Compostela. It is not the image of thinness that counts for these young girls (or not only), but (also) what thinness reflects: the deprivation of food is achieved at the cost of physical suffering, the taming of the bodily impulses (distancing from one’s animal nature, carnal pleasures) and of course self-discipline and tenacity. In the anorexic mind, the “excess” pounds correspond to the consequences of a vulgar behavior, which must be purged from the body through the intermediary of the discipline of fasting. The body is sculpted, not (solely) to correspond to an ideal of beauty, but to make it the exterior (perhaps visible, but not necessarily) of one’s monumental and grandiose interior. The body is the meeting point, the fusion, between morality and esthetics. The anorexic subject weakens their body to expose the purity of their interior. According to Nietzsche, asceticism is a sort of treatment to cure a “sick” existence: “(…) *the ascetic ideal has its source in the prophylactic instinct of a degenerating life which seeks to be cured, which, by any means, endeavours to preserve itself, which struggles for existence; it is the sign of depression and partial physiological exhaustion, against which arise unceasingly the deepest and most intact instincts of life, with constantly renewed inventions and artifices”* (Nietzsche, 2000).

It is striking to note to what extent Nietzsche’s ideas find an echo in the testimony of Marya Hornbacher, herself a former anorexic, taken from her autobiography (cited in Merand and Lemoine, 2022): *“I was incredibly tired of myself. I wanted to achieve this grandiose thing that was expected of me, whatever it was, […] and get it over and done with. To be able to sleep. […] I think it’s important to point out that food disorders are probably a cultural and generational version of good old burnout. […] I had not the slightest idea what I would do with myself once I had achieved “success,” but I could not turn my back on the frantic need to achieve it. […] People suffering from eating disorders tend to be both intelligent and competitive. We are terrible perfectionists. […] We are obsessed with […] trying to seem impressive. […] I become exhausted from the feeling of being constantly on stage, wearing someone else’s clothes, reciting someone else’s script.”*

We may stress the impact on others of the image of the emaciated body (which is apparent even in the emaciated traits of the face). There has always been throughout history a fascination for this extraordinary will-power, in particular among philosophers as Nietzsche noted: *“There exists a real concern, a particular affection among philosophers regarding the ascetic ideal.”* The historian R. Bell established a parallel between a medieval fast and modern day eating disorders in his book, *Holy Anorexia* (Bell, 1987). He described pious women in the Middle Ages as *“anorexic saints”* (Bell, 1987; cited in Darmon, 2003). In this context, modern day anorexia (at least certain of its forms) may be seen as a non-religious spiritual enterprise with the goal of being, an extravagant passion consisting in renouncing everything to become everything.

We may note that the rate of anorexia suddenly doubled in the USA between 1960 and 1975, that is in a transitional period when society rapidly became more individualistic (ideal of the self) with the baby-boomers and exceptional economic growth (Ehrenberger, 2018). This observation echoes the reflections of Nietzsche who spoke of epidemics of what might be compared to “melancholic” or “depressive” disorders: *“Man has had enough, often real epidemics occur of this satiety of living: but even this disgust, this languor, this self-contempt — all that assails him with such violence that he is reborn straightaway with new ties.”* It is not insignificant that anorexia is so prevalent in the USA, in particular among the wealthier classes (Darmon, 2003; Castel, 2009). This rich, developed country is the driver of the world capitalist system, consumption is unbridled and its model for success is the ‘self-made man’ referred to above. In American society, more than anywhere else in the world, the lower class is often associated with poor diet and thus with obesity. The fast-food chains are everywhere and any attitude involving eating healthily and in moderation has very positive connotations. In this context of overconsumption accessible to all (or almost all), values related to food are reversed and the dominant class achieves “the sublime” by restraint (frugality), the quality of food and slimness (Darmon, 2003; de Saint Pol, 2010): “*The process of civilisation described by Norbert Elias and which originally explained the evolution of corporal manifestations such as spitting or table manners may also apply to the form of the body. One of the reasons for the new attention to weight since the beginning of the 20th century might well be the wish to define new criteria of distinction which separate the wealthiest individuals from the coarseness and sloppiness of the poorest.”* (de Saint Pol, 2010) We are here at the opposite pole from the popular valorisation of the “*bon vivant*,” the overweight, the pot-bellied [(Hoggart, 1970) cited by Darmon (2003)]: *“With the representation of the self in the mode of taking oneself in hand and working on the body is indeed associated an explicit contempt for “letting yourself go” which is manifested at once in eating practices and the bodies of others. The fact of eating (and in particular eating ravenously, or making food something “convivial”) is taken as contemptible “vulgarity”* (Darmon, 2003). Anorexia is also very present in certain sports such as rhythmic gymnastics and dancing (Bratland-Sanda and Sundgot-Borgen, 2013). The ideology that may predominate is that a thin body may facilitate the performance of complex figures, that would be inaccessible or more difficult for a heavier body. But in fact the fleshless body has other meanings. It is the visible proof not only of the will-power of the sports-person, but also of their conformity to the customs of their chosen discipline. To integrate the sporting community, the young gymnast feels obliged to show solidarity with the privations of her partners. She would be guilty of not abiding by the rules (of privation) in force when others are making so much effort to succeed.

Anorexia arises from the meeting point between the human will and the questioning of the meaning of life on one hand, and the injunctions of society on the other. Humans, “*the being of the eternal future*” (Nietzsche, 2000) find themselves faced with the great question of the meaning of life, whose pertinence and form have changed incessantly during the course of history. This is how Nietzsche concludes the last part of the Genealogy of Morals on the ascetic ideal (Nietzsche, 2000): *“And this is the meaning of any ascetic ideal: he meant that something was missing, that a vast lacuna surrounded Man — he did not know how to justify himself, to interpret himself, to stand up for himself, he suffered before the problem of the meaning of life. (…) The meaningless of pain, and not pain itself, is the malediction that until today has weighed upon humanity — yet the ascetic ideal gives him a meaning! It has been until now the only meaning that he had been given; any meaning is better than no meaning at all; the ascetic ideal was from any point of view merely the “better than nothing”* par excellence*, the sole last resort there was. Thanks to it, suffering had found an explanation (…). The interpretation that might be given of life led undeniably to a new suffering, deeper, more intimate, more venomous, more deadly (…) But despite everything — it brought humanity salvation, Man had a meaning, he was now no longer the leaf blowing in the wind, the plaything of mindless chance, of meaninglessness, he could now wish for something, — little matter what he wished for, why, how rather such a thing than another: at least the will itself was saved. Impossible to dissimulate the nature and the meaning of the will to which the ascetic ideal had given direction: this hatred of what is human, and more still of what is “animal,” and more still what is “matter”; this horror of the senses, of reason itself; this fear of happiness and of beauty; this desire to flee all appearance, change, fate, death; effort, desire itself — all that means, let us dare to understand it, a desire for nothingness, a hostility to life, a refusal to accept the fundamental conditions of life; but it is at least, and will always be, a desire! … And to repeat once again and by finishing what I said at the beginning: Man still prefers to have a desire for nothingness than not to desire anything at all ….”*

Anorexia in the clinical sense, that is to say diminishing and imprisoning the subject in a destructive decline, closing them off from the world and from plans, and of which there is a wide range of causes, ultimately relies upon classical conditioning. Food gradually takes on negative connotations for the subject, for their entourage and for society (sport club, family ideal of health and of beauty, etc.), for the reasons outlined above. All food takes on a significance of impurity and all fatty matter becomes the sign of the imperfect taming of the body. Self-control becomes the rule and the goal, while thinness represents its visible face. It is this behavior which is valorized, hunger disappears, food becomes repulsive. The entourage who are worried at the situation can contribute to this conditioning by forcing the subject to eat, which adds yet another negative emotion to the situation. We observe that the implementation of this conditioning is similar to what prevails for misophonia. The two conditions share loyalty to certain social rules, in particular the imperative of discipline, rigor, self-control and no doubt also self-fulfillment. It is in addition interesting to note that anorexia is associated, like misophonia, with the personality trait of perfectionism (Lloyd et al., 2014). A few cases have been reported in the literature presenting the two disorders (Kluckow et al., 2014). This testimony from the book by Muriel Darmon illustrates this parallel between the two conditions (Darmon, 2003): *“Sometimes seeing my parents eating seemed almost obscene*. *I could not bear to watch them eating, it’s…[…] I do not know, it was pretty much gluttony personified, in fact … Well, my father is not very discreet anyway when he’s eating […]. There’s something very voracious that I do not like. Anyway it’s clear […]. Knowing all the others […] are…vulgar in the everyday sense, well, that their reactions, that their life is vulgar….”*

In short, we suggest that anorexia and misophonia share points in common: repugnance for “vulgar” sloppiness, lack of self-control and rigor. The two disorders might be the result of an excessively rigorous conditioning of the social rule, consisting in accumulating value by restraint and “polite” behavior. Nevertheless, the two disorders should not be confused. Anorexia is above all an enterprise turned toward oneself, consisting in improving, perfecting or perhaps honing oneself. Misophonia is on the contrary directed toward others and their rule violations. Whereas the response of anorexia to the vulgarity of the world is to surpass oneself through extreme discipline, misophonic subjects suffer from a diminished tolerance of the coarse behavior of others.

## General conclusion

In this article, we have attempted to present an explanation of the origin of misophonia. This article should be seen as an essay, a reflection under construction, and not as the presentation of a firm and definitive model. As with any reflection, what is developed in this article will evolve and be amended. Furthermore, this article should be seen as providing a different perspective on misophonia from that of the cognitive sciences. The latter divide the mind into major executive functions (working memory, action control, planning, attention, etc.) and can, through this reading grid, attribute the origin of a disorder (misophonia, for example) to a dysfunction in one or more of these functions (Eijsker et al., 2019; Daniels et al., 2020; Frank et al., 2020; Abramovitch et al., 2024). Our approach is not necessarily in complete contradiction with this model but attempts to account for the mind in a deeper, and more global and structural way, and from the point of view of the subject’s lived experience. In this case, we suggest that misophonia is the result of a disturbance in the relationship to the world, or in the organization of the world, where a sensory element (acoustics), failing to be integrated into the harmonious perceived world, is a source of turbulence. We also believe that psychology has everything to gain by distancing itself from positivism and its fetishism (or even idolatry) of the fact, without abandoning the empirical and scientific approach: “*A fact is a fact” (…). Focusing entirely on the fact as such, on the what, they never question the mode of access to the fact, the “how of its aim.” (…) The consequence of this narrowness of reason, which is also its accomplishment, is the risk of a partial knowledge or a truth separated from the whole being brought to the absolute,* i.e.*, ultimately a fetishized and therefore abstract knowledge*” (Husserl, 1997). We hope that the ideas and concepts presented and developed here will encourage debate and cultivate new approaches.

Briefly, we argue that human beings are shaped by social forces (respect, politeness, prestige, etc.), that these forces act on a mind that has its own structures (will, openness to the world, symbolic function, order) and mechanisms (learning and conditioning). As far as social forces are concerned, the pressure of modern society values individualization, autonomy, self-fulfillment and, at the same time, self-control and order in the perceived world, which can lead in extreme cases to an exaggerated sensitivity to disorder, immorality and vulgarity. The disorder may appear gradually, starting from a repeated situation (probably often a family one) that is difficult to escape or avoid (the evening meal), through the mechanisms of conditioning. Misophonic triggers, which have already acquired a negative connotation (through initial social conditioning and/or thought structure), may actually become misophonic triggers after further (often social) conditioning. The emotional reactions are generalized and spread (with very few exceptions) to other situations and people producing trigger sounds. We have established a parallel between misophonia and anorexia: it would appear that the two disorders have in common an excess of self-control and sensitivity to vulgarity (and the list of everything that is synonymous: trivial, common, coarse, ordinary; mediocre, rustic, crude, uncouth, etc.).

The question arises in this field of knowing whether this disorder should join the long list of mental disorders already included in the DSM (Diagnostic and Statistical Manual of Mental Disorders) (Schröder et al., 2013). It seems to us that any diagnosis, in particular that of including misophonia in the DSM, is based on an attempt at normalization. A frontier is established between “the normal and the pathological” (Canguilhem, 2013) and the disorder is positioned on one side or the other of this frontier. But this assessment grid for mental illness is not without significance: it consigns misophonia to a specific field (into the “dominion” of psychiatry) with its own methods and conceptions. Misophonia is then neither considered not treated in the same way. These issues of categorization between “the normal and the pathological” concerning psychological disorders are very complex, and we feel it would be dangerous to tip a condition such as misophonia into the domain of psychiatry. There is always to a certain extent a power struggle between scientific disciplines, notably medical and psychological, since their normative power is not only huge but is also subject to interpretation. One might argue about whether diabetes (stabilized) is a disease or not [in the sense of Canguilhem (2013)], but it would always be more difficult to establish a clear distinction for mental disorders. The first difficulty is the fact that behaviors are dynamic processes strongly influenced by the social rules. It is probable that modern day humans would all have been misophonic in the Middle Ages! And what would they have seemed like if they got upset at the violent slaughter of animals? There is certainly a whole range of psychological suffering (ill-being) which is crystallized in a certain form (anorexia, post-traumatic stress, misophonia, etc.) and which depend on “social forces” (Castel, 2009). These “forces” are notably the cause of the development of an interiority and a heightened sensitivity to what should be suppressed or at least hidden. It is more urgent to open up the disciplines, to decompartmentalize them, and to attempt to understand human beings through the interrelations between the different groups that study them, rather than biasing the debate by imposing from the outset a certain number of conceptual norms.

I leave to Norbert Elias the task of concluding: *“And we also come to a point where a straight road leads us to knock down the artificial borders which serve to divide up our reflections on humankind among the* var*ious disciplinary fields, that of the psychologist, that of the historian, and that of the sociologist. The structures of human interiority and that of history are indissociable complementary phenomena which can only be studied on the basis of their interdependence. They neither exist nor evolve in reality, any of them, in as independent a way as current research would have us believe. On the contrary, they constitute with other structures the subject of a single and unique science of humankind.”*

## Data availability statement

The original contributions presented in the study are included in the article/supplementary material, further inquiries can be directed to the corresponding author.

## Author contributions

The author confirms being the sole contributor of this work and has approved it for publication.
